# Case Report: Late-Onset Congenital Adrenal Hyperplasia and Acute Covid-19 Infection in a Pregnant Woman: Multidisciplinary Management

**DOI:** 10.3389/fendo.2020.602535

**Published:** 2021-01-15

**Authors:** Claudia Giavoli, Enrico Iurlaro, Valentina Morelli, Giulia Rodari, Andrea Ronchi, Carlo Pietrasanta, Lorenza Pugni, Daniela Tubiolo, Paolo Properzi, Antonio Pesenti, Giovanna Mantovani, Enrico Ferrazzi, Maura Arosio

**Affiliations:** ^1^Endocrinology Unit, Fondazione Istituto di Ricerca e Cura a Carattere Scientifico (IRCCS) Ca’ Granda Ospedale Maggiore Policlinico, Milan, Italy; ^2^Department of Clinical Sciences and Community Health, University of Milan, Milan, Italy; ^3^Unit of Obstetrics and Gynecology, Department of Woman, Child and Neonate, Fondazione IRCCS Ca’ Granda Ospedale Maggiore Policlinico, Milan, Italy; ^4^Neonatology and Neonatal Intensive Care Unit (NICU), Fondazione IRCCS Ca’ Granda Ospedale Maggiore Policlinico, Milan, Italy; ^5^Department of Anesthesia, Critical Care and Emergency, Fondazione IRCCS Ca’ Granda Ospedale Maggiore Policlinico, Milan, Italy; ^6^Department of Pathophysiology and Transplantation, University of Milan, Milan, Italy

**Keywords:** pregnancy, Covid-19, congenital adrenal hyperplasia, adrenal, steroid replacement

## Abstract

**Background:**

The impact of the Covid-19 infection on patients with chronic endocrine disease is not fully known. We describe here the first case of a pregnant woman with Covid-19 acute infection and non-classical congenital adrenal hyperplasia (NCAH).

**Case description:**

A woman at 36 weeks of gestation was referred to our Maternity Hospital for premature rupture of membranes (PROM). Her medical history was positive for NCAH on chronic steroid replacement till the age of 17 years (cortisone acetate and dexamethasone, both in the morning). At admission, her naso-oro-pharyngeal swab resulted positive for SARS-CoV-2. Due to hyperpyrexia and late preterm PROM, cesarean section was planned, and she was started on a 100 mg-bolus of hydrocortisone, followed by continuous infusion of 200 mg/24 h. A female neonate in good clinical condition and with a negative nasopharyngeal Covid-19 swab was delivered. On second *postpartum* day, the mother was in good condition and was switched to oral steroid therapy. On third *postpartum* day she worsened, with radiological signs of acute pulmonary embolism. Oro-tracheal intubation and mechanical ventilation were started, and she was switched back to intravenous steroid therapy. On April 30, pulmonary embolism was resolved, and on May 13th she was discharged in good condition.

**Conclusions:**

We report the first case of Covid-19 acute infection that occurred in late-pregnancy in a woman with NCAH on chronic steroid replacement. The management of the patient in a reference center with early involvement of a multidisciplinary team granted prompt care and adequate protection for all the involved sanitary operators.

## Background

In Italy, Lombardy was one of the first and worst hit regions by the novel coronavirus disease (Covid-19). Here we describe the first case of acute Covid-19 infection in a pregnant woman affected with late-onset congenital adrenal hyperplasia.

## Case Description

On April 6, 2020, a 39 years-old pregnant woman at 36 weeks of gestation was referred to our Maternity Hospital for premature rupture of membranes (PROM). In the previous days, she had suffered from hyperpyrexia up to 38.6°C, easily managed by paracetamol. Prior to admission, the naso-oro-pharyngeal swab performed in the emergency room resulted positive for SARS-CoV-2 at rRT-PCR assay. She weighed 97 kg, her height was 166 cm, blood pressure was 128/74 mmHg, O2sat was 100%, and respiratory rate was 16 bpm. Medical history was positive for late onset congenital adrenal hyperplasia due to 21-hydroxilase deficiency (NCAH) diagnosed at the age of 17 because of elevated basal 17-hydroxyprogesterone (17OHP) concentrations at the investigations performed for hyperandrogenism and alopecia. Since then, she was on steroid replacement therapy (cortisone acetate 12.5 mg plus dexamethasone 0.75 mg per day, both in the morning). She had two previous pregnancies, one six years earlier, delivered at term and one first trimester miscarriage. Glucocorticoid dosage had never been adjusted. Cesarean section was planned due to persisting hyperpyrexia and late preterm PROM. She was started on a 100 mg-bolus of hydrocortisone, followed by continuous infusion of 200 mg/24 h as indicated to avoid adrenal crisis in surgical intervention of chronic adrenal insufficient patients (1–3). She was delivered by cesarean section under spinal anesthesia, provided its positive influence on cardiopulmonary function (4).

At 36 weeks of gestational age, a female neonate was delivered. She was in good clinical condition, and her nasopharyngeal swab for SARS-CoV-2 by rRT-PCR was negative.

**Figure 1:**
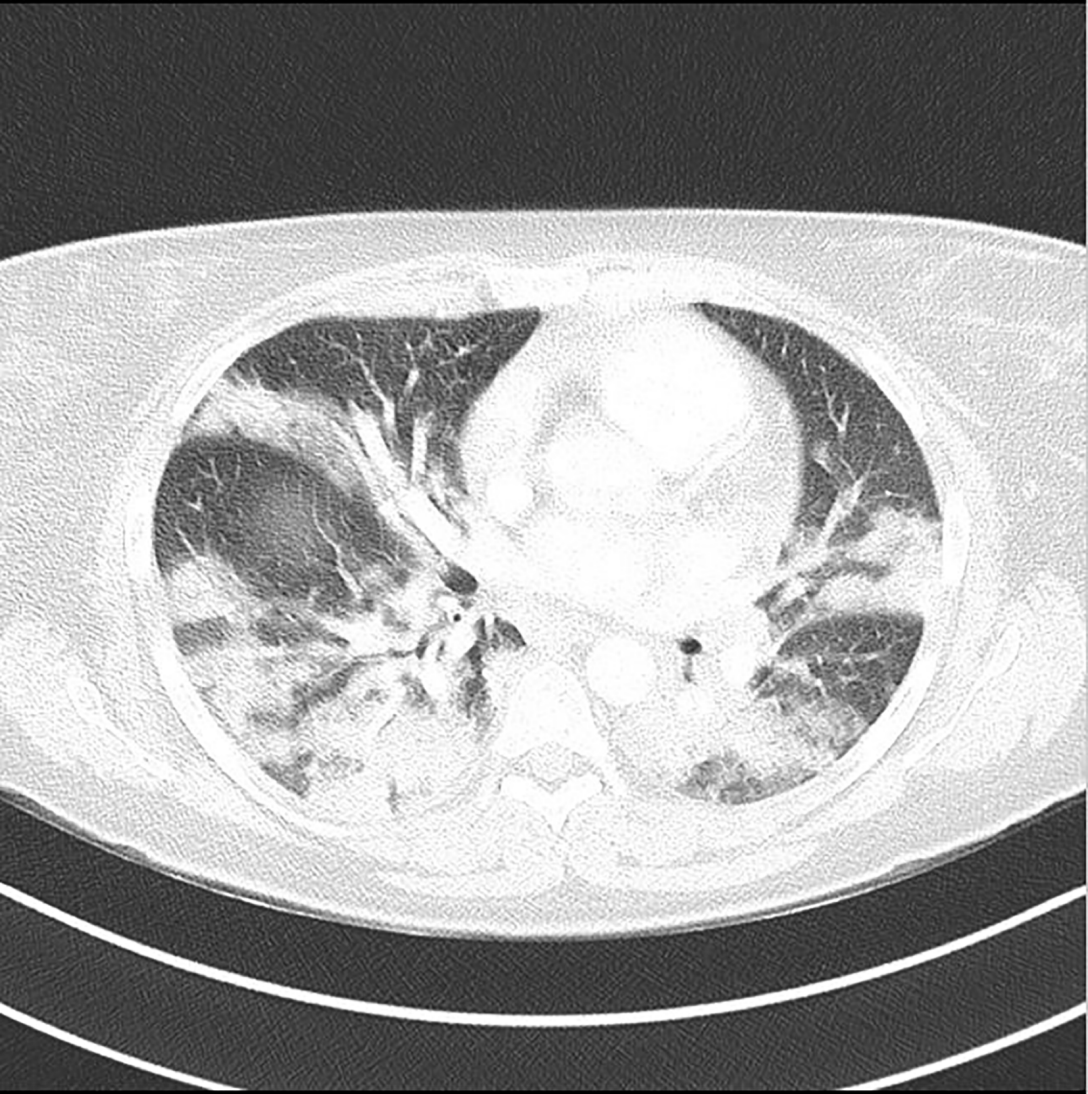
Chest CT showing signs of acute pulmonary embolism, along with extensive ground-glass opacifications involving both the lung parenchyma.

On second *postpartum* day, the mother was in good condition and was switched from intravenous to oral supra-physiological steroid therapy (cortisone acetate 25 + 12.5 + 12.5 mg/day); at that time since her temperature was 37.5°C, she was started on full dose low molecular weight heparin. On the third day her clinical conditions progressively worsened, with fever >39°C associated with respiratory symptoms requiring O2 support. Thus, therapy with hydroxychloroquine and azithromycin was started. Accordingly, cortisone acetate was increased to overall 75 mg/day. A Chest Computed Tomography (CT) showed signs of acute pulmonary embolism, along with extensive ground-glass opacifications involving both the lung parenchyma (**Figure 1**) and laboratory tests showed high D-Dimer (4,136 µg/L, nv <500 µg/L) and CRP (11.22 mg/dl, nv < 0.05 mg/dl). Her conditions rapidly worsened so she was moved to the Intensive Care Unit (ICU); orotracheal intubation along with mechanical ventilation were started, and she was promptly switched back to intravenous steroid therapy (Hydrocortisone, 50 mg every 6 h iv).

This aggressive respiratory and medical support allowed her to recover and, after the first week, to improve significantly her general condition. On April 30, pulmonary embolism was completely resolved at CT scans. Finally, on May 13th, she was discharged in good condition.

At the beginning of May, hydrocortisone was progressively reduced and switched to oral route (cortisone acetate 12.5 + 12.5 + 6.25 mg) to continue monitoring at our tertiary outpatient’s clinics.

## Discussion

We report the first case of Covid-19 acute infection that occurred in late-pregnancy in a woman with NCAH on chronic steroid replacement.

Non-classic congenital adrenal hyperplasia is an autosomal recessive disorder due to a deficiency of 21-hydroxylase, an essential enzyme for the adrenal synthesis of cortisol and aldosterone. NCAH patients typically have 20–70% residual enzyme activity, enough to maintain a normal electrolyte homeostasis by aldosterone, but with a variable deficiency of cortisol, reduced pituitary feed-back, and increased ACTH and adrenal androgen production. By suppressing ACTH production, glucocorticoids can normalise the excessive androgen production and can be used in symptomatic patients to increase fertility and during pregnancy (3, 5–7). However, during pregnancy and especially in NCAH, most experts do not recommend dexamethasone, a category C drug that passes the placenta and has potential adverse effects on both mother and fetus (3, 6, 8).

In the absence of data about her native adrenal function, we managed successfully the patient as adrenal insufficient according to the current Guidelines (1–3). Definitely, this could be assumed at least as resulting from long-term (more than 20 years) high dose steroid treatment.

The COVID-19 infection, in this case probably of familiar origin, affected many young women between March and April in Lombardy, where about 9% of cases occurred in women between 30 and 39 years (9). However, the disease was particularly severe in our patient requiring a long recovery in ICU with a particularly challenging management, since the infection spread in late pregnancy.

Indeed, a systematic review including 538 pregnant women with Covid-19 infection reported approximately the same rate of ICU admission as in the non-pregnant population, but an increased risk of preterm and cesarean delivery (10).

To frame the situation, in the period between March 1^st^ and April 30^th^, 852 women delivered in Mangiagalli Obstetrics Unit, a high-risk maternity center in Milan, and one of the six Covid-19 hubs identified in Lombardy to centralize care of pregnant patients affected by SARS Co-v 2 infection (11). Among these, 25 had confirmed COVID-19 infection (3%). In the Covid-19 group, 11 (44%) women delivered by cesarean and 14 (56%) vaginally. Only two women were admitted to the ICU and one in the sub-intensive Unit. The patient here described was the only one requiring mechanical ventilation.

It is possible to hypothesize that the long-term steroid therapy may have contributed to cause either a major risk of infection or a greater clinical picture severity. Concordant to this hypothesis, a recent paper reported an increased risk of lower respiratory tract infections in CAH patients on chronic glucocorticoids therapy, not only compared to general population, but also to CAH patients not treated with glucocorticoids, thus enlightening that non-physiological delivery of glucocorticoid replacement may represents a risk factor for infections development (12). Moreover, as in other critical illnesses, Covid-19 pneumonia can affect residual adrenal function through cytokine release, not only worsening the outcome but also raising the risk either of medical complications as well as of progressing to worse critical stages (13). Few data on Covid-19 infection spread and course in patients with adrenal disorders are available so far. A recent survey published by our group reported a similar rate and severity of Covid-related symptoms in adrenal insufficient patients and in controls (14). In this study CAH patients were excluded because of the possible detrimental role both of androgen excess and of not fully physiological steroid therapy.

While present report was under review, a first case of SARS-CoV-2 infection in a 5-week-old infant with adrenal insufficiency secondary to CAH was published (15). The authors underline the positive clinical course of SARS-CoV-2, even in the presence of underlying adrenal insufficiency and suggest that even though hydrocortisone was started to treat adrenal insufficiency, it may have also contributed to improve response to SARS-CoV-2 infection.

Similarly, in the woman here reported, hydrocortisone 200 mg/die (1) has been sufficient not only to prevent acute adrenal crisis but also to reach disease remission (16).

From the pandemic outspread up to now, plenty of literature has tried to widen the knowledge of this novel Coronavirus, and the medical approach is continuously evolving.

For instance, considering the latest data about the use of dexamethasone (17, 18), it is possible to hypothesize that an earlier use of higher corticosteroid dose might have prevented the worsening of her clinical conditions.

Similarly, even though no consensus is still available, more recent case series or reports about *postpartum* patients with Covid-19 infection tend to support the benefits of thromboprophylaxis (19). Moreover, in the last few months the role of immune response on the pathogenesis of Covid-19 complications pointed out the possible benefit of immune-modulators as adjuvant therapy (20). In this context, reports about the use of melatonin suggest its possible beneficial effects in anti-inflammation, anti-oxidation, immune response regulation, as demonstrated in respiratory disorder models induced by infections. Thus, even though the direct evidence of melatonin application in Covid-19 is not fully clear, given also its high safety profile, these suggestions may prompt its use in Covid-19 patients (21).

Treating an acute Covid-19 infection, spread in late pregnancy and at the beginning of Covid-19 pandemic, was particularly challenging not only for the adrenal condition, but also for all the uncertainties on best therapeutic approaches.

Undoubtedly, the management of the patient and the neonate in a reference center with early involvement of a multidisciplinary team, through application of the best medical knowledges available at that time, granted prompt care for them and adequate protection for all the involved sanitary operators.

## Data Availability Statement

The raw data supporting the conclusions of this article will be made available by the authors, without undue reservation.

## Ethics Statement

Ethical review and approval was not required for the study on human participants in accordance with the local legislation and institutional requirements. The patients/participants provided their written informed consent to participate in this study. Written informed consent was obtained from the individual(s) for the publication of any potentially identifiable images or data included in this article.

## Author Contributions

CG, EI, VM, CP, AR, CP, LP, DT, and PP performed treatment and follow-up of the patients and the newborn. CG, VM, and GR collected clinical data and prepared the manuscript. AP, GM, EF, and MA performed the critical revision of the manuscript. All authors contributed to the article and approved the submitted version.

## Conflict of Interest

The authors declare that the research was conducted in the absence of any commercial or financial relationships that could be construed as a potential conflict of interest.
